# Timing mechanisms to control heart rhythm and initiate arrhythmias: roles for intracellular organelles, signalling pathways and subsarcolemmal Ca^2+^

**DOI:** 10.1098/rstb.2022.0170

**Published:** 2023-06-19

**Authors:** Derek A. Terrar

**Affiliations:** Department of Pharmacology, University of Oxford, Oxford OX1 3QT, UK

**Keywords:** heart rhythm, calcium, arrhythmia, sarcoplasmic reticulum, endolysosome, mitochondria

## Abstract

Rhythms of electrical activity in all regions of the heart can be influenced by a variety of intracellular membrane bound organelles. This is true both for normal pacemaker activity and for abnormal rhythms including those caused by early and delayed afterdepolarizations under pathological conditions. The influence of the sarcoplasmic reticulum (SR) on cardiac electrical activity is widely recognized, but other intracellular organelles including lysosomes and mitochondria also contribute. Intracellular organelles can provide a timing mechanism (such as an SR clock driven by cyclic uptake and release of Ca^2+^, with an important influence of intraluminal Ca^2+^), and/or can act as a Ca^2+^ store involved in signalling mechanisms. Ca^2+^ plays many diverse roles including carrying electric current, driving electrogenic sodium–calcium exchange (NCX) particularly when Ca^2+^ is extruded across the surface membrane causing depolarization, and activation of enzymes which target organelles and surface membrane proteins. Heart function is also influenced by Ca^2+^ mobilizing agents (cADP-ribose, nicotinic acid adenine dinucleotide phosphate and inositol trisphosphate) acting on intracellular organelles. Lysosomal Ca^2+^ release exerts its effects via calcium/calmodulin-dependent protein kinase II to promote SR Ca^2+^ uptake, and contributes to arrhythmias resulting from excessive beta-adrenoceptor stimulation. A separate arrhythmogenic mechanism involves lysosomes, mitochondria and SR. Interacting intracellular organelles, therefore, have profound effects on heart rhythms and NCX plays a central role.

This article is part of the theme issue ‘The heartbeat: its molecular basis and physiological mechanisms’.

## 1. Introduction

Previous reviews provide discussions of the cellular and intercellular mechanisms that give rise to arrhythmogenic events (e.g. [1–7]) and it is widely accepted that arrhythmias are a major health problem (e.g. [8–11]). The focus of this review is on diverse roles of a variety of intracellular organelles in initiating or influencing the timing of electrical activity in myocytes throughout the heart, including their importance in the generation of arrhythmias. These organelles include the sarcoplasmic reticulum (SR), mitochondria and organelles in the lysosome family.

**Figure 1.**
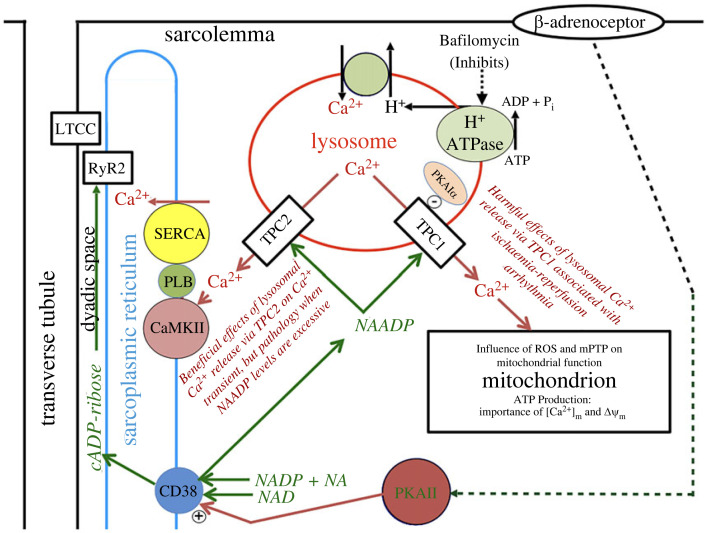
Diagrammatic representation of relative locations of SR, lysosomes and mitochondria in a ventricular myocyte. The lysosome is shown between the SR and the mitochondrion. The action of H^+^-ATP-ase generates an acidic lumen, and this acidity drives Ca^2+^ accumulation by a H^+^–Ca^2+^ exchange mechanism. Bafilomycin inhibits lysosome function by inhibiting the H^+^-ATP-ase. The enzyme CD38 is shown here as located on the SR (see text) and catalyses the synthesis of both nicotinic acid adenine dinucleotide phosphate (NAADP; by a base exchange reaction involving NADP and nicotinic acid, NA) and cADP-ribose (by an ADP-ribosyl cyclase reaction involving NAD). NAADP causes Ca^2+^ release from the lysosome via two-pore channel 2 (TPC2), and this Ca^2+^ activates Ca^2+^/calmodulin-dependent protein kinase II (CaMKII) to phosphorylate phospholamban (PLB) and, therefore, enhance Ca^2+^ uptake into the SR by SERCA. This effect is beneficial, unless the stimulation is excessive. NAADP also acts to cause Ca^2+^ release from the lysosomes via two-pore channel 1 (TPC1) and this Ca^2+^ interacts with mitochondria to provoke an arrhythmia resulting from reperfusion following ischaemia. Reactive oxygen species (ROS) and mitochondrial transition pore (mPTP) are particularly important in these arrhythmias. PKAIalpha is thought to be protective by blocking TPC1. ATP production is shown as being controlled by the Ca^2+^ concentration in the mitochondrial matrix ([Ca^2+^]_m_) and the voltage across the inner mitochondrial membrane (Δ*Ψ*_m_). cADP-ribose causes additional Ca^2+^ release from the SR via ryanodine receptor type 2 (RyR2s) by an action from the cytosol. The precise site of action of cADP-ribose remains controversial (see text). Beta-adrenoceptor stimulation causes enhanced synthesis of both cADP-ribose and NAADP by CD38. The L-type Ca^2+^ channels (LTCC) are shown in the transverse tubular membrane located close to RyR2s in the SR membrane, and separated by the dyadic space. The novel signalling pathways shown here are in addition to the well-known β-adrenoceptor actions via protein kinase A (PKA) on LTCC, PLB/SERCA and RyR2s [12]. (Online version in colour.)

**Figure 2.**
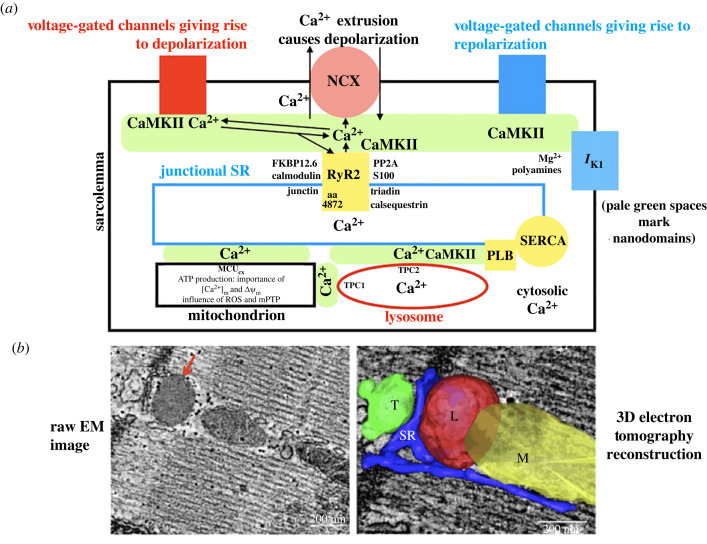
(*a*) A general scheme for proteins controlling electrical activity of heart cells. Ion channel proteins in the surface membrane play a primary role (grouped together as playing either a depolarizing or repolarizing influence, see text), but interacting intracellular organelles also exert important regulatory actions under particular conditions, and these influences can be linked to changes in membrane potential, particularly when Ca^2+^ is released from an organelle into one of the nanodomains involved in cell signalling (shown as pale green spaces). These organelles include SR, lysosomes and mitochondria. The SR is shown with the primary Ca^2+^ uptake mechanism via SERCA (regulated by PLB which can be phosphorylated by both PKA and CaMKII), while Ca^2+^ release is primarily via RyR2. Both cytosolic and luminal factors influence opening of RyR2s, and the amino acid at position 4872 is at a crucially important luminal site for regulating Ca^2+^ release. Sodium–calcium exchange protein (NCX) is shown as centrally important since it links changes in subsarcolemmal Ca^2+^ to membrane depolarization when it is extruding Ca^2+^. Subsarcolemmal Ca^2+^ is increased by Ca^2+^ entry via L-type Ca^2+^ channels in the surface membrane and by Ca^2+^ release from the SR via RyR2s. The mitochondrial uniporter complex (MCU_cx_), is shown in the mitochondrial membrane. ATP production is shown as being controlled by the Ca^2+^ concentration in the mitochondrial matrix ([Ca^2+^]_m_) and the voltage across the inner mitochondrial membrane (Δ*Ψ*_m_). ROS and mPTP are particularly important in arrhythmias (see text for details). (*b*) Electron microscopy (EM) images illustrating the relative locations of transverse tubules, SR, lysosomes and mitochondria. The red arrow in the raw EM image on the left indicates the lysosome. On the right is a three-dimensional electron tomography reconstruction with the transverse tubule (T) shown in green, the SR blue, the lysosome (L) red and the mitochondrion (M) yellow. Images adapted from Aston *et al.* [13]. (Online version in colour.)

Interactions between organelles and the functional nanodomains between them are shown schematically in figures 1 and 2 before presenting detailed evidence in subsequent sections.

Changes in membrane potential across the sarcolemma are controlled by the operation of a variety of ion channels and electrogenic ion transporters. An influence of intracellular organelles on these channels and transporters requires a functional linkage between them. It will be argued below that electrogenic sodium–calcium exchange (NCX) plays a key role in this linkage (figure 2).

Organelles can directly contribute a timing mechanism (such as an SR clock driven by cyclic uptake and release of Ca^2+^, probably involving intraluminal Ca^2+^ concentration reaching a critical level to control this release), and/or can provide an intracellular store for Ca^2+^ involved in signalling mechanisms. The release of Ca^2+^ from intracellular organelles is central to the control of most if not all cell functions [14–16], and the heart is no exception.

The SR will be discussed first, since it exerts a dominant role as the major Ca^2+^ store [17–20]. Lysosomes, a component of the endolysosomal system, will be considered next since accumulating evidence shows that these organelles are important throughout the body with functions that depend on Ca^2+^ release [14,16,21–26], and it is becoming recognized that lysosomes also have regulatory functions in the heart [27]. Mitochondria are widely distributed throughout all types of cardiac myocyte and are vital in providing an energy supply for heart function [28,29]. Although under most circumstances their direct influence on electrical activity is surprisingly small the effects of mitochondria can become dominant under particular conditions including ischaemia followed by reperfusion when lysosomes also play a role.

Another theme of this review concerns evidence for interactions between organelles. These interactions are controlled by calcium mobilizing agents which regulate cellular activity in many organ systems of the body [16]. Such agents have received limited attention in the context of the heart, although they are discussed in a recent review [12]. Calcium mobilizing agents include nicotinic acid adenine dinucleotide phosphate (NAADP), cADP-ribose and inositol trisphosphate (IP_3_) [12,30]. Evidence will be discussed showing that cardiac function, including electrical activity, is modulated by each of these calcium mobilizing agents acting on intracellular organelles. Pathways for NAADP and cADP-ribose are shown schematically in figure 1. Enzymes activated or inhibited by Ca^2+^ also make contributions to the integration of functions. Ca^2+^/calmodulin-dependent protein kinase II (CaMKII) is well known as a regulator of cardiac activity [31], but more recent evidence discussed below highlights additional roles for CaMKII in signalling pathways where interactions between intracellular organelles are mediated by calcium mobilizing agents. This additional evidence enables an improved understanding of the influence of CaMKII on electrical activity.

It will be argued here that timing mechanisms associated with the initiation of major arrhythmias in atrial and ventricular myocytes (including those arising from afterdepolarizations associated with the action potential (AP)) share many of the same features as those underlying the natural pacemaker activity of cells of the sinoatrial (SA) and atrio-ventricular (AV) nodes.

## 2. Locations of intracellular organelles and the importance of nanodomains

This section provides an overview to guide the reader in the following detailed discussion. Experimental evidence is kept to a minimum but is fully explained in the appropriate later sections. Figure 1 shows a schematic of the location and interaction of intracellular organelles, including pathways indicating operation of the calcium mobilizing agents, NAADP and cADP-ribose. The figure omits the membrane proteins crucial for supporting electrical activity which are emphasized in figure 2. Figure 1 also shows the position of the transverse tubule in a ventricular myocyte with embedded L-type Ca^2+^ channels (LTCC). The adjacent SR contains ryanodine receptor type 2 (RyR2) for Ca^2+^ release and sarcoplasmic/endoplasmic reticulum Ca^2+^-ATPase (SERCA) (regulated by phospholamban (PLB)) for Ca^2+^ uptake. The central role of CaMKII, fulfilling multiple functions, is discussed in the following section. Lysosomes are shown between SR and mitochondria ([13,27] and figure 2*b* for electron microscopy (EM) evidence). Mitochondria also make close connections with SR and Ca^2+^ can exert effects in both directions. CD38 catalyses synthesis of both NAADP and cADP-ribose. In the heart, CD38 has been observed to be very close to the SR and it is possible that it is located within the SR membrane as shown here (although this is not yet fully established and is controversial). Synthesis of both NAADP and cADP-ribose is enhanced following stimulation of the beta-adrenoceptor. NAADP initiates lysosomal Ca^2+^ release via two-pore channels (TPCs). Ca^2+^ release via TPC2 channels acts on SR while Ca^2+^ release via TPC1 channels acts on mitochondria. Evidence supports an inhibitory influence of PKARIalpha on TPC1. cADP-ribose is shown acting on RyR2s but this is again controversial. Lysosomes are acidic and the pH gradient is generated by a vacuolar H^+^-ATP-ase. Ca^2+^ accumulation in the lysosome is dependent on the pH gradient, probably via H^+^–Ca^2+^ exchange in the lysosomal membrane. Bafilomycin inhibits vacuolar H^+^-ATP-ase and is a very useful experimental tool, since the collapse of the pH gradient suppresses accumulation of Ca^2+^ within lysosomes [32,33]. Bafilomycin, therefore, abolishes both the ability of the lysosome to take up Ca^2+^ and any functional effects that depend on lysosomal Ca^2+^ release. Reactive oxygen species (ROS) and the mitochondrial transition pore (mPTP) are important for mitochondrial function and will be discussed in the context of the relevant arrhythmias.

Figure 2*a* complements figure 1 by emphasizing the importance of nanodomains between different organelles. Nanodomains are shown as pale green spaces. These nanodomains permit Ca^2+^ released from organelles to achieve high local concentrations to influence neighbouring cell components. Again the scheme shows SR, lysosomes and mitochondria. Key proteins in the SR include RyR2s to release Ca^2+^ and PLB/SERCA to control Ca^2+^ uptake from the cytosol to the lumen of the SR. PLB can be phosphorylated by both protein kinase A (PKA) and CaMKII. Also shown are calsequestrin, junctin and triadin which exert a luminal influence on RyR2. FKBP12.6 and calmodulin will be considered below. Discussion of the importance of PP2A and S100 which are a thought to modulate RyR2 on the cytosolic side is beyond the scope of this review but can be found in [6]. Amino acid number 4872 in RyR2 is emphasized because at this site luminal Ca^2+^ exerts a particular influence on RyR2 opening (see text in section on delayed afterdepolarizations (DADs) and [34]).

Figure 2*a* also shows proteins in the surface membrane which normally control electrical activity. A key theme of this review is the importance of NCX in linking activity of organelles to changes in membrane potential. A rise in subsarcolemmal Ca^2+^ occurs when Ca^2+^ is released from the SR. The increase in subsarcolemmal Ca^2+^ in turn leads to Ca^2+^ extrusion via NCX accompanied by membrane depolarization. The magnitude of Ca^2+^ released from the SR can be increased when more Ca^2+^ is stored within the SR. Greater uptake of Ca^2+^ into the SR can occur as a result of lysosomal activity. This arises when NAADP causes Ca^2+^ release from the lysosomes via TPC2 channels into a nanodomain between the lysosome and the SR. The mechanism involves activation of CaMKII by the rise in Ca^2+^ in this nanodomain leading to phosphorylation of PLB and increased activity of SERCA, therefore increasing SR Ca^2+^ uptake.

Since NCX is fundamentally important both for natural pacemaker activity and for the initiation of arrhythmogenic events it is given a separate section below. The central position of NCX in figure 1 reflects this importance.

**Figure 3.**
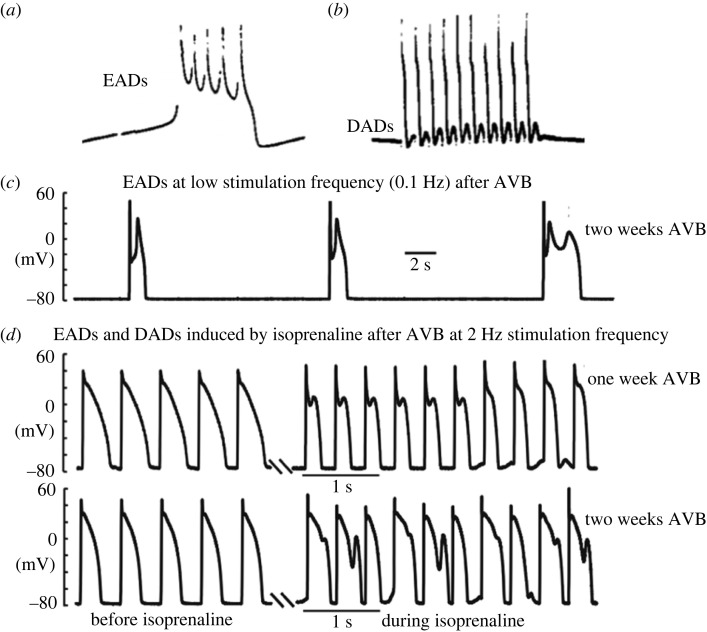
(*a*,*b*) Early records of early and delayed afterdepolarizations (EADs and DADs; adapted from Cranefield [35] and Ferrier & Moe [36]) recorded from Purkinje fibres. EADs in A arise in the region of the plateau of the AP, while DADs in B occur close to the resting potential. (*c*) Action potentials in ventricular myocytes from rabbits with chronic atria-ventricular block (AVB) causing downregulation of K^+^ channels. At low rates of stimulation EADs were triggered at potentials close to the plateau. (*d*) Records at a higher rate of stimulation before and after beta-adrenoceptor stimulation with isoprenaline. In the presence of isoprenaline, many APs show associated EADs, and there are also DADs close to the resting potential. Some events occur at potentials where the distinction between EADs and DADs is less clear. Records adapted from Qi *et al.* [37].

In addition, figure 2*a* shows voltage-gated ion channels with a depolarizing or repolarizing influence collected together to simplify discussion. In Purkinje fibres, ventricular and atrial myocytes, the major voltage-gated channels with a depolarizing influence are Na_V_1.5 carrying *I*_Na_, Ca_V_1.2 carrying *I*_Ca_. In these cell types the major voltage-gated channels with a repolarizing influence are hERG carrying *I*_Kr_ and KCNQ1 with KCNE1 carrying I_Ks_ (and in most species K_v_4.2, K_v_4.3 and K_v_1.4 carrying *I*_to_ just after the AP peak). It is beyond the scope of this review to provide a detailed discussion of how opening and closing of ion channels and activation of electrogenic mechanisms give rise to different AP waveforms in different regions of the heart. Examples of ventricular waveforms are shown in figures 3*d*, 5*a* and 6*a*. A pacemaker waveform from an SA node myocyte is shown in the top trace of figure 7*b*. In general terms, activation of voltage-gated channels giving rise to depolarization causes the upstroke of APs, and the membrane potential is restored by activation of another set of voltage-gated channels giving rise to repolarization. The different waveform shapes can be accounted for by variations in the activation, inactivation and de-activation of the various ion channels in the different cell types. Details of the underlying mechanisms for different waveforms can be found in [38] for ventricle, in [39] for atria, in [40] for SA node and in [41] for AV node.

*I*_K1_ is shown separately in figure 2*a* since although it is not voltage activated in a conventional sense, its ability to conduct K^+^ ions is nevertheless greatly modified by depolarization, and it is a major determinant of membrane potential in atrial and ventricular myocytes. While open, *I*_K1_ channels exert a dominant influence to hold the membrane potential close to the resting level, but the depolarization brought about by activation of Na^+^ channels largely removes this influence since it leads to channel blockade by intracellular Mg^2+^ and polyamines ([42–45] and see [46]). Consequently at membrane potentials close to the plateau when *I*_K1_ channels are predominantly blocked in ventricular myocytes, the repolarizing influence of *I*_K1_ is lost and in addition the effects of depolarizing currents are enhanced (because by simple Ohm's law, *V* = *IR*, effects, the increase in membrane resistance (*R*) when *I*_K1_ channels are blocked by Mg^2+^ or polyamines means that the same depolarizing current, *I*, has a greater effect on membrane potential, *V*). It will become apparent that this behaviour of *I*_K1_ is particularly important in the context of differences between early afterdepolarizations (EADs) and DADs.

In the SA node (and AV node), a major factor determining spontaneous activity is lack of *I*_K1_, so that there is no maintained negative ‘resting’ potential, and the effective membrane resistance is relatively high, enhancing effects of individual currents.

Figure 2*b* shows EM records of the junctions between the organelles shown schematically in figure 2*a*. A red arrow marks the lysosome in the raw EM image. The three-dimensional electron tomography reconstruction shows a transverse tubule (green), SR (blue), lysosome (red) and mitochondrion (yellow). Note the network arrangement of the SR forming junctions with both the lysosome and mitochondrion in addition to the transverse tubule.

Under pathological conditions components of the network summarized in figures 1 and 2 that might initially be dormant or negligible may show a substantial increase in importance.

## 3. The importance of Ca^2+^/calmodulin-dependent protein kinase II

As shown schematically in figure 2, CaMKII is a key intermediary in bringing about effects of Ca^2+^ in nanodomains. Actions of CaMKII include modification of LTCC, RyR2s and PLB/SERCA [47–50]. In figure 2, CaMKII is shown close to the relevant targets to reflect these functional effects. In the case of LTCC, CaMKII causes a facilitation involving an increase in amplitude and a slowing of decay [51,52]. The action of CaMKII on RyR2s is to enhance Ca^2+^-induced-Ca^2+^ release [53,54]. Phosphorylation of PLB by CaMKII leads to enhanced activity of SERCA to promote Ca^2+^ uptake into the SR. There are also effects of CaMKII on ion channels including *I*_Na_, *I*_to_ and *I*_K1_ (see [31,55] for reviews).

As a consequence of these many and diverse mechanisms, CaMKII is particularly important in arrhythmias, including effects on SR Ca^2+^ content [48,56,57]. CaMKII is also important for normal pacemaker activity of the SA node [50].

In addition to these well-known mechanisms it will become apparent from evidence presented below that CaMKII is also important in the actions of NAADP-mediated Ca^2+^ release from lysosomes and for effects of cADP-ribose on SR Ca^2+^ release. The significance of these mechanisms for the initiation of arrhythmias will be considered again in an additional section on CaMKII at the end of this review.

## 4. The central role of sodium–calcium exchange in the timing mechanisms which determine heart rate and initiate arrhythmias

NCX is given a short separate section here since its importance in the timing mechanisms controlling electrical activity is a recurring theme throughout this review. As noted in relation to figure 2 NCX is essential for normal pacemaker activity in SA and AV nodes, and in many cases it determines the initiation of arrhythmias. It is also an essential link between organelle function and electrical activity in the sarcolemma. NCX acts in so many different cellular contexts that detailed evidence for its contributions to the generation of different types of arrhythmia and to normal pacemaker activity will be discussed in later sections under appropriate separate headings.

The operation of NCX is reviewed in [58]. NCX is electrogenic since 3 Na^+^ are exchanged for each Ca^2+^, so that one excess charge crosses the membrane for each cycle [58].

Before discussing the role of NCX contributions in pacemaker and arrhythmogenic mechanisms, the contribution of NCX to the AP waveform will first be briefly considered for atrial and ventricular myocytes during ‘normal’ activity at a stable heart rate. NCX is expected to be active both during the AP plateau, and between beats to keep cytosolic Ca^2+^ at a low level of approximately 100 nM. During the AP Ca^2+^ entry via LTCC triggers further Ca^2+^ release from the SR via RyRs [19,20], generating a Ca^2+^ transient in which approximately two-thirds of the Ca^2+^ derives from the SR and the remaining one third results from Ca^2+^ entry across the surface membrane (at least in most mammalian species including human, see [30,59]). The Ca^2+^ transient drives the contraction essential for the pumping action of the heart. At body temperature the declining phase of the Ca^2+^ transient occurs before repolarization of the AP is complete (see example in figure 6*a*). The decline of the Ca^2+^ transient is determined by the competing influences of SERCA to take Ca^2+^ back into the SR and NCX to extrude Ca^2+^ across the sarcolemma. The precise timing of NCX is controversial but in the steady state the amount of Ca^2+^ extruded must be approximately equal to the amount of Ca^2+^ provided by the major influx pathway of LTCC. There may, however, be a period during the early part of the plateau when SERCA is so dominant that the approximately two-thirds of the Ca^2+^ contributing to the Ca^2+^ transient that is provided by SR Ca^2+^ release can be taken back up into the SR. While NCX is extruding the remaining third of Ca^2+^ it must exert a depolarizing influence, thus helping to support maintenance of the AP plateau. The precise timing of this contribution varies with species, and is discussed in [60].

When NCX contributes to arrhythmias, there is a major role for Ca^2+^ released from the SR, though recent evidence presented in subsequent sections shows that this is often modulated by the activity of other organelles. The importance of the SR will be discussed first.

## 5. The role of the sarcoplasmic reticulum in the generation of arrhythmias

### (a) Afterdepolarizations

One important source of disturbances to heart rhythm concerns afterdepolarizations that arise in association with the cardiac AP under abnormal or pathogical conditions in ventricular or atrial myocytes, and also in Purkinje fibres. EADs occur soon after the upstroke of the AP while the membrane potential is still elevated close to plateau potentials. DADs occur at a later stage in the AP waveform when repolarization from the plateau is well underway [35,61,62] (figure 3). In other words DADs are associated with what would normally be quiet periods in between APs, while EADs occur close to the normal plateau of the AP. These EADs and DADs were first described in Purkinje fibres [35,36], a widely used experimental preparation in early electrophysiological studies. Similar afterdepolarizations have been recorded in ventricular (figure 3*c* adapted from [37]) and atrial myocytes. Extracellular potassium ions and *I*_K1_ are particularly important in determining whether the membrane potential is closer to the plateau or the resting potential [46,63,64]. It is generally agreed that DADs result from additional SR Ca^2+^ release occurring soon after the primary Ca^2+^ transient triggered by the AP when repolarization is almost complete (figure 3), and detailed experimental support for this view will be discussed in the next section. After providing evidence for the central importance of SR in DAD mechanisms, the possible contribution of SR Ca^2+^ release to EADs, which is currently less well accepted, will also be considered.

### (b) The role of sarcoplasmic reticulum in delayed afterdepolarizations

Early work in papillary muscle from ferret heart [65] showed that DADs were suppressed either by ryanodine to block SR RyR2s, or by loading of the cells with the Ca^2+^ chelator BAPTA applied via the microelectrode to keep subsarcolemmal Ca^2+^ at a very low level. It was concluded that DADs were associated with Ca^2+^ release from the SR, and that the increased subsarcolemmal Ca^2+^ drove Ca^2+^ extrusion across the surface membrane by electrogenic NCX to produce the depolarizations.

Even before these observations, experimental evidence showed that cytosolic Ca^2+^ oscillations occurring under appropriate experimental challenges (beta-adrenoceptor stimulation or cardiac glycoside to promote the amount of Ca^2+^ loaded into the SR) were associated with membrane potential oscillations in single myocytes [66], cardiac Purkinje fibres [67] and ventricular muscle [68]. Later work in whole hearts also supports the above general hypothesis [69].

**Figure 4.**
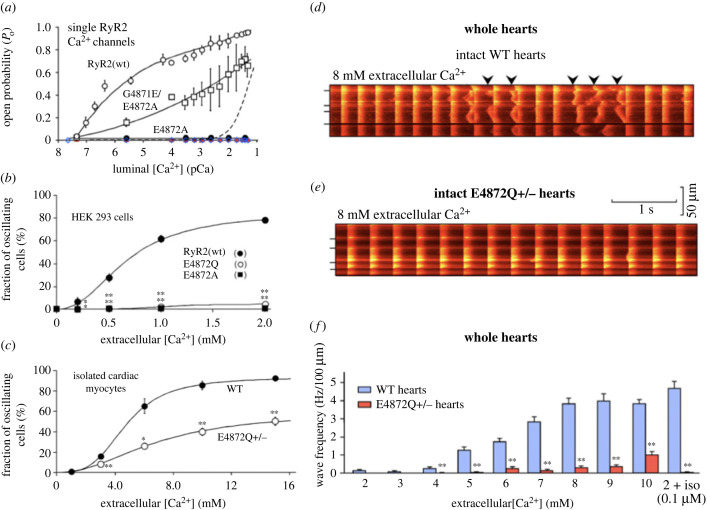
(*a*) Open probability (*P*_o_) of RyR2 channels in planar lipid bilayers with increasing luminal Ca^2+^ concentration. Note low sensitivity to luminal Ca^2+^ of E4872A channels compared with wild-type (WT). The graphs in (*b*) and (*c*) show the fraction of cells exhibiting spontaneous oscillations as the extracellular Ca^2+^ was increased. (*b*) HEK 293 cells expressing RyR2, and (*c*) ventricular myocytes. In comparison to WT, spontaneous activity was greatly suppressed by the RyR2 amino acid substitutions E4782A and E4782Q shown in (*b*). The ventricular myocytes in (*c*) are from mice with an RyR2 mutation suppressing SR luminal Ca^2+^ actions (one copy in heterozygous mice, E4872Q^+/−^); in the case of E4872Q^+/−^ the proportion of cells showing oscillations was approximately half that in WT, consistent with expectations for a heterozygous phenotype, with E4872Q showing minimal Ca^2+^ sensitivity. (*d*,*e*) Ca^2+^ traces recorded using confocal microscopy from whole hearts at a high extracellular Ca^2+^ concentration of 8 mM either in WT (*d*) or when RyR2 was modified in the heterozygous mice, E4872Q^+/−^, (*e*). Spontaneous activity is evident in the WT (with arrow heads indicating Ca^2+^ waves) but not in the heterozygous mice with modified RyR2. (*f*) A summary of observations in which the height of the bars shows the frequency of wave occurrence either in WT or E4872Q^+/−^ hearts, when extracellular Ca^2+^ was increased or during exposure to isoprenaline. It can be concluded that RyR2 modification at site 4872 has profound effects on spontaneous activity, while single channel studies in planar lipid membranes showed that the E4782A and E4782Q mutations greatly suppressed actions of SR Ca^2+^ at a luminal site. Records from Chen *et al.* [34]. (Online version in colour.)

The normal process triggering Ca^2+^ release from the SR involves Ca^2+^-induced-Ca^2+^-release in which Ca^2+^ ions act on the cytosolic face of RyR2s to trigger Ca^2+^ release through RyR2 channels [19,20,70]. However, the work on Ca^2+^ oscillations quoted in the previous paragraph supports the proposal that the SR can be ‘overloaded’ with Ca^2+^, giving rise to ‘store overload induced Ca^2+^ release’ [70–72]. It appears that opening of RyR2s also depends on Ca^2+^ within the lumen of the SR and there is a balance of influence between factors on both the cytosolic and luminal sides of the SR membrane. Experiments on isolated SR in planar lipid bilayers also support an influence of Ca^2+^ on the luminal side [73,74]. Genetic modification of RyR2s can suppress store overload induced Ca^2+^ release, providing convincing evidence that particular amino acids in the RyR2 structure are involved in this process ([34] figure 4). In experiments on planar lipid bilayers, a point mutation at position 4872 abolished RyR2 activation by luminal but not cytosolic Ca^2+^ (figure 4*a*). This substitution also greatly reduced the number of oscillating cells when extracellular Ca^2+^ was increased (figure 4*b*,*c*) or the beta-adrenoceptor agonist isoprenaline was applied. Substitution of another amino acid at the same site also caused abolition of sensitivity to luminal Ca^2+^ but was expected to produce a slightly less severe phenotype on the basis of single channel studies, and mice with a heterozygous form of this mutation survived (while the homozygous form was lethal at the embryonic stage). Whole hearts from heterozygous mice (with one copy of the gene coding for the RyR2 modification involved in luminal Ca^2+^ sensitivity) showed substantially reduced propensity to generate Ca^2+^ waves in response to either high extracellular Ca^2+^ or isoprenaline (figure 4*d–f*, [34]). The above evidence provides strong support for the importance of luminal Ca^2+^ in initiating Ca^2+^ waves by direct effects at a particular site on the RyR2. Another naturally occurring RyR2 mutation is responsible for catecholaminergic polymorphic ventricular tachycardia (CPVT) in humans, and myocytes from mouse hearts with the same mutation showed increased tendency for store overload induced Ca^2+^ release [75,76]. As expected these hearts showed CPVT in response to caffeine and adrenaline. Mouse hearts were generated with both the CPVT mutation and the above modification at site 4872 in the RyR2 which causes a substantial reduction of luminal Ca^2+^ sensitivity. Hearts from these hybrid mice did not show CPVT in response to caffeine and adrenaline, providing further evidence for the actions of luminal Ca^2+^ in these arrhythmias [34,77].

Although it seems that Ca^2+^ within the SR might reach a critical level to initiate release by direct action at the RyR2, it should be emphasized that other factors also influence Ca^2+^ release. During a DAD Ca^2+^ release is spontaneous in that it is not directly triggered by the normal excitation–contraction mechanism even though it is linked to the preceding APs. A key influence is uptake of Ca^2+^ into the SR via SERCA since this will determine luminal Ca^2+^ concentration. Ca^2+^ uptake by SERCA is in turn regulated by PLB, which can be phosphorylated by PKA or CaMKII ([78], figure 2).

**Figure 5.**
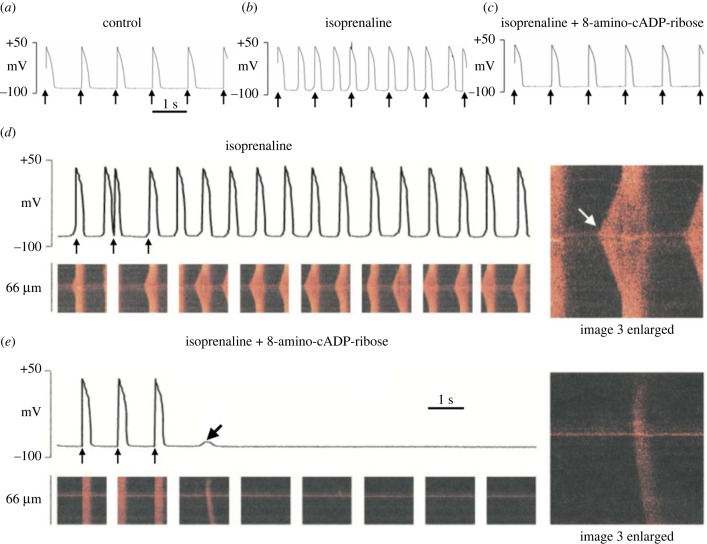
(*a–c*) The top rows show APs recorded from guinea pig ventricular myocytes, with arrows marking the time of stimulation. Addition of isoprenaline in (*b*) provoked additional spontaneous action potentials, which were suppressed in (*c*) by cytosolic application of 8-amino-cADP-ribose, an antagonist of the calcium mobilizing agent, cADP-ribose. (*d*) Action potentials (APs) in the presence of isoprenaline with corresponding Ca^2+^ transients recorded by linescan confocal microscopy. The first three vertical arrows below the APs mark the stimuli, so that the following APs are spontaneously generated at a remarkably constant rate. The linescans show Ca^2+^ waves, which gave rise to synchronized activity across the cell when the action potential was initiated. A magnified view of a Ca^2+^ wave and synchronized transient is shown on the right. (*e*) Records in the same cell after application of 8-amino-cADP-ribose. It can be seen that the spontaneous action potentials and Ca^2+^ waves were greatly suppressed, and only one low intensity wave remained giving rise to a small subthreshold depolarization. Again a magnified view of the wave is shown on the right. Adapted from Rakovic *et al.* [81]. (Online version in colour.)

The above effects involving SR, particularly those determined by the Ca^2+^ level within the lumen, may be exacerbated by an influence of lysosomes. Especially during beta-adrenoceptor stimulation, Ca^2+^ released from lysosomes acts on CaMKII to activate SERCA and further increase SR Ca^2+^ uptake ([79] reviewed in [27]). Again particularly during beta-adrenoceptor stimulation Ca^2+^ release can also be increased by the actions of the Ca^2+^ mobilizing agent cADP-ribose to enhance RyR2 sensitivity to cytosolic Ca^2+^ [30]. cADP-ribose actions also appear to depend on CaMKII [80]. In support of the contribution of cADP-ribose to arrhythmias, an antagonist of cADP-ribose suppressed arrhythmogenic Ca^2+^ oscillations provoked by a high concentration of beta-adrenoceptor agonist ([81], figure 5). These mechanisms are discussed in more detail below in the sections devoted to lysosomes and calcium mobilizing agents.

Additional factors influence Ca^2+^ release via RyR2s by actions at a cytosolic site. These include PP2A and S100 ([6] figure 2). Furthermore the RyR2 is subject to control by an array of luminal proteins shown in figure 2. These include calsequestrin, junctin and triadin, as discussed in [6,82–84].

The discussion above concentrates on DADs in ventricular muscle, but similar mechanisms arise in Purkinje fibres [85] and atrial muscle [86], even though there are important differences in the organization of the SR and transverse tubules in all three types of cardiac muscle [87,88].

Overall the above evidence shows that while cytosolic factors do influence the tendency for Ca^2+^ to be released from the SR, the process of triggering a DAD is primarily driven by factors within the SR, particularly the luminal Ca^2+^ concentration reaching a critical level.

### (c) Importance of sarcoplasmic reticulum for early afterdepolarizations in addition to other factors including ‘late’ L-type Ca^2+^ current

EADs have been much less well understood than DADs, and various mechanisms may make different contributions depending on precise conditions. In experiments quoted above in which ryanodine blocked DADs, EADs remained [65]. EADs were, however, suppressed when LTCC were blocked by nitrendipine. It was proposed that ‘persistent’ Ca^2+^ current during the prolonged plateau could support the observed membrane potential oscillations [65]. Extensive theoretical discussions conclude that ‘window’ current results from overlap of activation and inactivation curves for L-type Ca^2+^ currents. Computer modelling shows that these currents can support EADs [11,64]. The extent to which a component of L-type Ca^2+^ current persists during the late plateau of the AP remains difficult to establish by experimental methods but many recent studies continue to consider ‘late’ Ca^2+^ current as a major mechanism supporting EADs (e.g. [89]). Persistence of Ca^2+^ current is expected to lead to additional Ca^2+^ loading of the SR both by directly providing Ca^2+^ for SR uptake and by prolonging the plateau of the AP at potentials which favour Ca^2+^ uptake by the SR in competition with Ca^2+^ extrusion via NCX (less effective at depolarized potentials because of the reduced tendency for Na^+^ to enter). L-type Ca^2+^ currents also contribute directly to cytosolic Ca^2+^ which must then be removed, principally by electrogenic NCX. Blockade of LTCC would, therefore, result in suppression not only of window currents but also of additional Ca^2+^ loading of the SR and NCX currents resulting from extrusion of both the additional Ca^2+^ from the SR and that entering via the L-type Ca^2+^ currents.

There may also be a contribution of late Na^+^ current, as reviewed in [90]. As in the case of L-type Ca^2+^ currents, late Na^+^ current may arise as a consequence of overlap of activation and inactivation curves. Late Na^+^ current can be enhanced by drugs or mutations that delay inactivation. Recent experiments support arrhythmogenic effects of this pathway [91,92].

**Figure 6.**
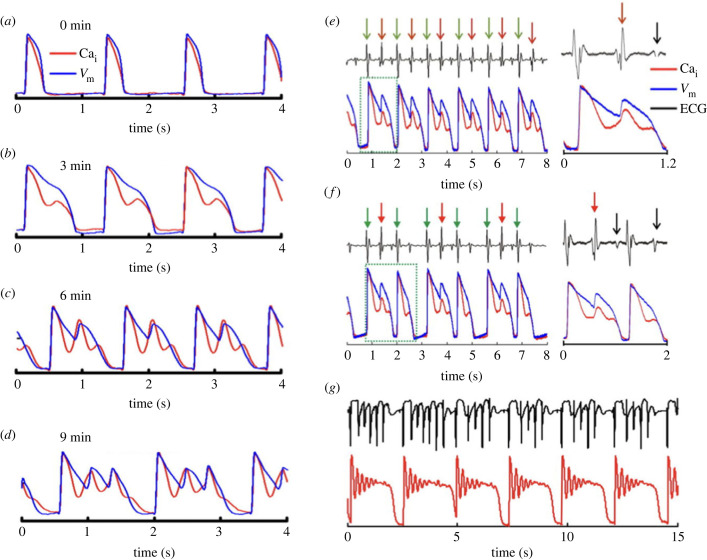
Recordings from Langendorff-perfused whole hearts, with blue traces representing membrane potential measured with RH237 and red traces show cytosolic Ca^2+^ measured with rhod-2. The records in (*a*) show that the Ca^2+^ transient rose very rapidly with only a very small lag following the upstroke of the AP, and re-uptake of Ca^2+^ occurred before full repolarization. EADs were provoked by blockade of *I*_Kr_ with dofetilide in (*c*,*d*) (blue traces). It can be seen that an additional rising phase of Ca^2+^ transient was evident in (*b*) even before EADs were obvious, and in (*c*) the secondary rise in Ca^2+^ (red trace) occurred before the start of each EAD. In (*d*), there were two EADs each preceded by a rise in Ca^2+^. (*e*,*f*) Further records during exposure to dofetilide similar to those in the previous panels, but with an accompanying electrocardiogram (ECG). Again EADs are evident, but the activity progressed to the development of ‘alternans’ with alternating long and short APs, and corresponding changes in the duration of the Ca^2+^ transient. Green arrows mark paced beats while red arrows show ventricular ectopic beats. Black arrows mark T waves. (*g*) shows simultaneous ECG and Ca^2+^ records with brief runs of polymorphic VT. Pacing rate was 50 bpm with 2 : 1 capture. Each run of polymorphic VT was associated with a single CaT with multiple secondary events. Records adapted from Nemec *et al.* [99]. (Online version in colour.)

Despite the early observation of EAD resistance to ryanodine, later studies have shown contributions associated with Ca^2+^ loading of the SR to generation of EADs [93–96]. In 2000, the need to revise our understanding of EADs and torsades de pointes arrhythmias was discussed [97], and it was concluded that subsarcolemmal Ca^2+^ and NCX needed to be taken into account. Subsequent studies of whole hearts using an optical probe for membrane potential and a fluorescent Ca^2+^ indicator showed that a rise in cytosolic Ca^2+^ could precede depolarizations associated with an EAD and EADs were, therefore, likely to result from SR Ca^2+^ release ([98,99]; figure 6). EADs were provoked by either E4031 or dofetilide to block *I*_Kr_. It was concluded that Ca^2+^ overload of the SR led to spontaneous release of Ca^2+^, and the rise in subsarcolemmal Ca^2+^ drove ‘calcium-activated currents' that included NCX and led to the initiation of the depolarization of the EAD. Cryoablation of Purkinje fibres failed to prevent the EADs and arrhythmias, and it was concluded that ventricular myocytes were at least as likely as cells of the conduction system to initiate EADs. It was said that ‘Salvos of EADs produced undulating ECG patterns associated with TdP (torsades de pointes arrhythmias), which progressed to VT.’

AP prolongation involving downregulation of delayed rectifier K^+^ currents has also been investigated in rabbit hearts with chronic AV block [37]. Ventricular myocytes from this model showed EADs associated with the prolonged APs (figure 3). Blocking SR Ca^2+^ uptake with thapsigargin and SR Ca^2+^ release with ryanodine both suppressed the EADs. Interestingly, the EADs were also suppressed by the calmodulin inhibitor W7 and the CaMKII inhibitor, KN-93 [37]. Possible effects of the CaMKII pathway to promote persistence of L-type Ca^2+^ currents were considered, but there was also evidence for increased Ca^2+^ loading of the SR. Whether activation of CaMKII might at least in part reflect involvement of lysosomes in enhancing Ca^2+^ loading of the SR and/or cADP-ribose actions on RyR2 is considered below since CaMKII activity has been shown to be necessary for these effects (see also [100]).

More recently Ca^2+^ ‘ripples’ and late Ca^2+^ sparks have been observed to be associated with later stages of ventricular APs [101]. A related study from the same group showed that in ventricular cells from failing hearts, EADs occurred during prolonged APs and were associated with late Ca^2+^ sparks [102]. These observations support contributions from SR Ca^2+^ release.

EADs can also occur in atrial myocytes [7].

### (d) Similarities and differences between early afterdepolarizations and delayed afterdepolarizations

It seems there are many similarities between EADs and DADs, particularly concerning the role of the SR. The main distinguishing feature between these two types of arrhythmia is the range of membrane potentials at which they arise. Residual L-type Ca^2+^ currents might persist during EADs and provide contributions to the overall mechanisms, perhaps involving window currents, but also including additional SR Ca^2+^ uptake [64]. In the previous section discussing origins of DADs, the underlying mechanisms included the possibility that intraluminal Ca^2+^ might reach a critical level, presumably acting at site 4872 on the RyR2 (together with other cytosolic and luminal factors including calsequestrin, junctin and triadin), and such a mechanism might also play a role during EADs in many, if not most, conditions in which the resulting arrhythmias occur.

In the context of comparing EADs and DADs it is worth mentioning again the crucial importance of *I*_K1_ in determining whether the membrane potential is close to plateau when SR Ca^2+^ release contributes to EADs or to the resting potential when SR Ca^2+^ release initiates DADs [46,63]. Both experimental and computer modelling approaches support the influence of *I*_K1_ on afterdepolarizations [64,103].

### (e) The importance of the sarcoplasmic reticulum in ‘alternans’

In addition to the importance of the SR for DADs and EADs, there are related mechanisms concerning Ca^2+^ handling during cardiac ‘alternans’, a condition in which there are alternating long and short APs associated with an accompanying alternation in the amplitudes of Ca^2+^ transients, usually large Ca^2+^ transients with long APs and small Ca^2+^ transients with short APs [104–109]. The experiments of Nemec *et al.* [99] show that EADs can progress to alternans (figure 6) and SR function is the determining factor. It is again expected that changes in subsarcolemmal Ca^2+^ will influence AP waveforms via NCX. Alternans is associated with arrhythmias [99,105–107,109].

### (f) Implications of altered Ca^2+^ handling for initiation and spread of arrhythmias in intact hearts

When DADs and EADs occur in cells or isolated tissue, questions arise concerning whether ‘focal’ activity of this kind could generate widespread cardiac arrhythmias. However, whole heart work of Salama and colleagues quoted above and more recent experiments applying confocal microscopy to whole hearts [110,111] show that these mechanisms can indeed be arrhythmogenic. The occurrence of cardiac alternans has also been shown to be associated with clinical arrhythmias [112,113]. Another aspect of the initiation and sustenance of cardiac arrhythmias is thought to involve ‘re-entrant' pathways’, perhaps involving rotating waves of electrical activity [114]. There may be important differences concerning fibrillation in ventricles and atria since it has been argued that the relatively thick ventricular tissue may support rotating waves in three dimensions, and this activity might be fundamentally different from two-dimensional activity in atrial muscle [114].

Advances in mapping electrical activity in whole human hearts hold enormous promise for a greater understanding of arrhythmias in a clinical setting [115]. Recent work highlights the importance of rotors and wavelets of electrical activity in ventricular fibrillation [116,117] and considers the involvement of the Purkinje fibre network [118,119].

## 6. Role of sarcoplasmic reticulum in pacemaking

Early work accounts for spontaneous activity in SA node with little or no mention of intracellular organelles, as in an excellent review [120]. Ironically observations on DADs in ventricular muscle paved the way for investigations of the possible role of the SR in influencing the pacemaker activity of the SAN. In early experiments on ventricular myocytes we noted that high concentrations of beta-adrenoceptor agonist isoprenaline first caused DADs which went on to initiate APs and prolonged exposure could result in regular spontaneous activity as shown in figure 5*d* [81]. Observations of this kind led us to test for the effects of ryanodine on SA node preparations, postulating that Ca^2+^ release from the SR might contribute to pacemaker activity by a variety of mechanisms including electrogenic NCX [121]. Ryanodine applied to multicellular atrial preparations containing the SA node caused a clear slowing of the regular spontaneous generation of APs. A concentration of ryanodine expected to cause complete blockade of SR Ca^2+^ release slowed beating rate by about 30%, but substantial pacemaker activity remained. This was the first indication that the electrical activity of the SA node pacemaker (and actions of ryanodine) might share some features with generation of EADs and DADs in atrial or ventricular muscle. Ryanodine had been suggested to influence subsidiary pacemaker cells [122], but there was no widespread view that this was part of the normal physiological mechanism of pacemaker activity in the SA node.

**Figure 7.**
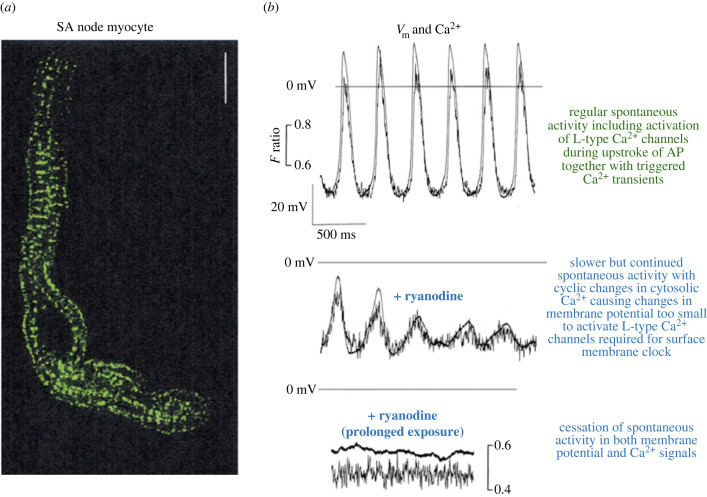
(*a*) A guinea pig pacemaker cell labelled with a fluorescent antibody to RyR2. The RyR2s are sparse but are located with the junctional SR at the edges of the cell just below the sarcolemma, and are also evident as clear striations corresponding to the arrangement of the non-junctional SR. (*b*) Top pair of traces shows records of membrane potential and cytosolic Ca^2+^ (noisy trace) measured with indo-1 in the presence of isoprenaline, which remained throughout the experiment. Horizontal line marks 0 mV for each pair of traces. The middle pair of traces are records from the same cell soon after application of ryanodine to block RyR2s causing a slowing of activity, but it should be noted that Ca^2+^ oscillations continued to occur at the reduced rate, even though the depolarizations were subthreshold for activation of Ca^2^ channels and, therefore, too small to support the surface membrane clock (see text). Further exposure to ryanodine under these conditions (bottom pair of traces) caused complete cessation of both Ca^2+^ and membrane potential oscillations. Note that Ca^2^ fluorescent probes, including indo-1, inevitably have Ca^2+^ buffering actions, and this may enhance the actions of ryanodine on rate. In the absence of indo-1 ryanodine slows but does not stop beating. Fluorescence calibration with bottom pair of traces also applies to middle traces. Adapted from Rigg *et al.* [123]. (Online version in colour.)

Further work in isolated pacemaker cells and multicellular atrial preparations showed the importance of ryanodine sensitive mechanisms both in the absence and presence of beta-adrenoceptor stimulation [123]. An informative observation was that when cytosolic Ca^2+^ was monitored with indo-1 (low concentration to minimize Ca^2+^ buffering effects, see below), ryanodine initially reduced Ca^2+^ transient amplitude while repetitive activity in the Ca^2+^ signal continued to occur at a reduced rate even though the accompanying changes in membrane potential were subthreshold for activation LTCC (figure 7). This would be consistent with a timing mechanism involving the SR alone.

In subsequent work over more than 20 years, the contribution of SR mechanisms to natural pacemaker activity in SA and AV nodes has been the subject of extensive research [123–130], with particularly helpful work on amphibian pacemaker activity [131–133]. Crucial experiments from the Lakatta group [125] showed that when a beating pacemaker myocyte was voltage-clamped at a negative potential (so that regenerative electrical activity in the surface membrane was prevented) rhythmic oscillations in cytosolic Ca^2+^ continued to occur at a rate similar to the original spontaneous APs. It is now widely accepted that the SR at least contributes to pacemaker activity [128,134]. In relation to DADs (and EADs), evidence showed that the timing of SR Ca^2+^ release might be determined by intraluminal Ca^2+^ reaching a critical level, and this may also be true for natural pacemaker activity. The timing mechanism involving the SR is sometimes referred to as a ‘Ca^2+^ clock’ [127] but since Ca^2+^ also plays such a central role in the surface membrane pacemaker mechanisms, I would prefer to use the terms SR clock and surface membrane clock. Recent research demonstrates the complexity of Ca^2+^ dependent mechanisms [135].

Continuing research on pacemaker mechanisms emphasizes roles for subtypes of Ca^2+^ channels, so that Ca_V_1.2 thought to support the AP upstroke can be supplemented by Ca_V_1.3 contributing to slow pacemaker depolarization [136]. Ca_V_1.3 is also involved in ‘sustained inward current’ (*I*_st_). *I*_CaL_ and *I*_st_ are said to share Ca_V_1.3 as a common molecular determinant [137]. *I*(f) currents contribute to the surface membrane clock [138,139], but even under conditions in which blockade of *I*(f) is close to complete (with a combination of blockers, ZD7288 and ivabradine [140]) pacemaker depolarizations are still maintained by a combination of deactivating voltage-gated K^+^ channels, Ca_V_1.2, Ca_V_ 1.3, background current and NCX [141], together with the lack of stabilizing influence of *I*_K1_. Interestingly, components of the membrane clock other than *I*(f) seem to be able to maintain beating even in the absence of the SR clock. This was demonstrated by the persistence of spontaneous beating during block of *I*(f) with ZD7288 followed by subsequent addition of ryanodine to prevent SR Ca^2+^ release via RyR2s or cyclopiazonic acid to prevent Ca^2+^ uptake into the SR by SERCA [140]. NCX currents extruding Ca^2+^ entry resulting from L-type Ca^2+^ currents would be expected to remain under these conditions.

Another intracellular mechanism to be included involves Ca^2+^ activation of Ca^2+^-stimulated adenylyl cyclases leading to cAMP production and protein phosphorylation including membrane ion channels [140,142,143]. Support for this suggestion is the observation that the SERCA antagonist cyclopiazonic acid caused a rapid reduction in Ca^2+^ transient amplitude, followed by a slow reduction in rate while Ca^2+^ transient amplitude remained constant, perhaps reflecting a slow suppression of actions of cAMP and PKA on membrane targets [140].

The importance of subsarcolemmal Ca^2+^ driving NCX-mediated depolarizations was emphasized above in the context of DADs and EADs. Such currents are also an essential component in the SR clock, since NCX provides the crucial linkage between changes in subsarcolemmal Ca^2+^ concentration resulting from cyclic SR Ca^2+^ release and changes in membrane potential. Even in the absence of SR function, NCX drives the surface membrane clock since cyclic increases in subsarcolemmal Ca^2+^ also result from Ca^2+^ entry through LTCC in the surface membrane. This leads to cyclic Ca^2+^ extrusion via NCX and accompanying depolarizations. Since NCX is crucial for both SR and surface membrane clocks, it is not surprising that blocking NCX causes complete cessation of pacemaker activity in isolated SA node cells [144]. Cytosolic application of the Ca^2+^ chelators BAPTA and EGTA also completely blocked spontaneous electrical activity, and this was thought to result primarily from chelation of the Ca^2+^ required to activate the depolarizing NCX currents [144]. Excessive cell loading with fluorescent Ca^2+^ probes which inevitably have Ca^2+^ buffering effects also causes cessation of electrical activity [123].

The above hypothesis and experimental work demonstrating the central importance of NCX was given further support in experiments in mice with a complete atrial-specific knockout of NCX protein [145]. Although atria were quiescent, these animals survived, perhaps as a result of generation of impulses in the AV node or another part of the conduction system. Interestingly, Ca^2+^ activity still occurred in the SR of SA node cells isolated from mice with the atrial-specific NCX knockout, but there was a complete loss of initiation of spontaneous electrical activity in the surface membrane [145,146].

The observations in the previous paragraphs, therefore, support the fundamental importance of NCX for pacemaker activity in the SA node. This conclusion shows a parallel with the discussion of DADs and EADs in earlier sections.

## 7. Lysosomes

Organelles in the endolysosome family have many diverse functions [21,32,33]. It is difficult to distinguish subtypes, particularly by light microscopy. For consistency the term lysosome will be used here to denote acidic organelles that are involved in intraorganelle signalling, particularly involving lysosomal effects on SR and mitochondria. One role concerns a contribution to the normal process of control of the amplitude of Ca^2+^ transients and contractions associated with APs. Excessive stimulation of this lysosomal pathway initiates arrhythmias. A second separate pathway involves interactions between lysosomes and mitochondria in a particular type of arrhythmia which results from reperfusion after a period of ischaemia. Both mechanisms are reviewed in [27], and see [12]. The transformation of Ca^2+^ release from the lysosome into electrical activity at the surface membrane depends on the SR via mechanisms that are at least in part similar to those discussed above. Evidence for lysosomal function is presented below after discussing the location of lysosomes.

### (a) Location of lysosomes

EM studies showed that lysosomes in the heart are approximately 400 × 300 nm [13]. In ventricular myocytes, lysosomes were strategically placed near both the SR and mitochondria enabling Ca^2+^ signalling in nanodomains between these organelles. The median separation was 20 nm in the case of SR, and 17 nm for mitochondria [13]. 3D tomography studies of these observations (figure 2*b*) show that the proximity may be even closer, 3.3 nm for SR and 6.2 nm for mitochondria.

By light microscopy it is difficult to distinguish different subtypes of acidic organelles. Lysotracker has been used in fluorescence light microscopy to identify lysosomes and related organelles since it accumulates in acidic stores. Lysosomes identified in this way are present in both ventricular [147] and atrial [148] myocytes and are organized in a punctate pattern. Bafilomycin (figure 1) prevents uptake of lysostracker, and suppresses functional effects resulting from lysosomal Ca^2+^ release. Lysosomes have also been shown to be present in cardiac ventricular myocytes using fluorescence microscopy and LAMP-2 antibodies [13]. The binding shows a periodicity along the long axis of the myocytes which is similar to that for antibodies to RyR2 and PLB, as expected for proximity to SR. Lysosomes identified with LAMP-2 were closely aligned but not colocalized with RyR2 and PLB. Ned-19, a fluorescent NAADP antagonist, binds to lysosomes identified by LAMP-2 antibodies and the binding was prevented by another NAADP antagonist, BZ194 [13]. Lysosomes were also observed using stimulated emission depletion microscopy and LAMP-2 antibodies, with a spatial resolution greater than conventional fluorescence microscopy. These studies showed that lysosomes are less than 1 µm in diameter [149], confirming the above EM observations.

### (b) Actions of nicotinic acid adenine dinucleotide phosphate to release Ca^2+^ from lysosomes, and subsequent effects on Ca^2+^ transients

In many cell types NAADP acts as a Ca^2+^ mobilizing agent provoking Ca^2+^ release from lysosomes [14,16,21,150–152], though there may be a binding protein that mediates the effects ([33] see discussion in [27]). Whether these effects are direct or not, photorelease of NAADP from a caged compound increased the amplitude of Ca^2+^ transients accompanying APs in guinea pig ventricular myocytes [147]. These effects were prevented by prior exposure to bafilomycin. The increase in Ca^2+^ transient amplitude in guinea pig ventricular myocytes was associated with increased uptake of Ca^2+^ into the SR, assessed by application of high concentrations of caffeine. There was no effect of photoreleased NAADP on the amplitude of L-type Ca^2+^ currents which trigger Ca^2+^ release from the SR. NAADP can also be applied as an acetoxymethyl (AM) ester, entering the cell so that intracellular esterases liberate NAADP [147]. Application of NAADP-AM increased the amplitude of contractions accompanying APs, and these effects were again prevented by bafilomycin. NAADP-AM increased the frequency and amplitude of Ca^2+^ sparks in rat ventricular myocytes [147]. The increase in Ca^2+^ spark amplitude further supports an effect of NAADP on Ca^2+^ uptake to increase SR Ca^2+^ load. Effects of NAADP-AM on Ca^2+^ spark frequency and amplitude were prevented by bafilomycin.

Ca^2+^ release from lysosomes in a variety of cell types has been shown to be mediated by ion channels formed from two subunits each with two pore domains [153–155]. In heart, TPCs occur in two varieties, TPC1 and TPC2. The two channels show differences in gating and ion conducting properties (although both show selectivity for Ca^2+^) and may play complementary roles [156]. Some have questioned the selectivity of TPCs for Ca^2+^, and in addition these channels can be activated by both NAADP and PI(3,5)2. These controversies have been extensively discussed in a recent review [33]. The bulk of the experimental evidence shows that Ca^2+^ is a major component of NAADP-mediated ion efflux via lysosomal TPC2 channels, and the presence of lipid enhances Ca^2+^ selectivity [27,33]. The selectivity of TPC1 channels may be lower [33] but the significance of this difference in a cardiac muscle context has yet to be explored. NAADP-AM caused increases in the amplitudes of Ca^2+^ transients accompanying APs in wild-type (WT), but not in ventricular myocytes lacking TPC2 channels, supporting the requirement of lysosomal Ca^2+^ release via TPC2 channels for the effects observed in WT myocytes [79]. TPC1 channels will be discussed later.

### (c) The contribution of lysosomal Ca^2+^ release to the effects of beta-adrenoceptor stimulation on Ca^2+^ transients accompanying action potentials

Under physiological conditions, actions of NAADP become particularly important during beta-adrenoceptor stimulation since this increases synthesis of endogenous NAADP [147,157]. In cardiac myocytes an early observation was that desensitizing or self-activating effects of high concentrations of NAADP (see [158]) to decrease the amplitude of contractions accompanying APs were greater in the presence than in the absence of the beta-adrenoceptor agonist isoprenaline [147]. In ventricular myocytes and whole hearts effects of isoprenaline on calcium transients accompanying APs or contractions were approximately one third less in TPC2 KO than in WT [79]. There was no difference between myocytes from TPC2 KO and WT in the increase in amplitude of L-type Ca^2+^ currents following exposure to isoprenaline.

Similar conclusions were drawn from observations in guinea pig ventricular myocytes using bafilomycin to suppress NAADP actions, or by addition of the NAADP antagonist Ned-19 [79]. Effects of isoprenaline on the amplitude of calcium transients accompanying APs were again approximately one third less in the presence than in the absence of drugs. Ned-19 did not reduce the effect of isoprenaline on the amplitude of L-type Ca^2+^ currents.

The observations above are consistent with the proposal that approximately one third of the overall actions of beta-adrenoceptor stimulation resulted from effects of NAADP acting via TPC2 channels in lysosomes. The remaining effects of beta-adrenoceptor stimulation presumably resulted from well-known effects on L-type Ca^2+^ currents, RyR2s and PLB/SERCA [78].

As discussed above, effects of NAADP on lysosomes are thought to be associated with increased SR Ca^2+^ load. In guinea pig ventricular myocytes the increase in amplitude of Ca^2+^ transients accompanying APs following photorelease of NAADP was prevented by autocamtide inhibitor peptide, a highly selective CaMKII inhibitor. The increase in amplitude of Ca^2+^ transients accompanying APs in mouse ventricular myocytes exposed to NAADP-AM was also prevented by the CaMKII inhibitor KN-93 [79]. The EM anatomical evidence shows a nanodomain between lysosomes and SR (separation approx. 20 nm). The observations are, therefore, consistent with NAADP actions to release Ca^2+^ from lysosomes into a nanodomain next to SR to stimulate CaMKII phosphorylation of PLB, with the result that SERCA-mediated uptake of Ca^2+^ into the SR is promoted.

Similar effects of NAADP occur in atrial myocytes. Both photorelease of NAADP and application of NAADP-AM caused an increase in the amplitude of Ca^2+^ transients accompanying APs [79,148]. Again the effects result primarily from an enhanced uptake of Ca^2+^ into the SR since high concentrations of caffeine caused a greater release of Ca^2+^ from the SR after NAADP photorelease [148], and the amplitude of Ca^2+^ sparks was increased after application of NAADP-AM [148]. The CaMKII inhibitor, KN-93, prevented the effects of both photoreleased NAADP and NAADP-AM [79]. The effects were suppressed by bafilomycin. Photorelease of NAADP did not increase the amplitude of L-type Ca^2+^ currents in atrial myocytes.

As was the case in ventricular myocytes, the effects of the beta-adrenoceptor agonist isoprenaline to increase the amplitude of Ca^2+^ transients in atrial myocytes was reduced by bafilomycin, and also by another agent, glycl-L-phenylalanine 2-napthylamide, which causes osmotic lysis of lysosomes [148].

### (d) Excessive activation of Ca^2+^ release from lysosomes via two-pore domain channel 2 is associated with arrhythmias

Arrhythmias discussed in earlier sections above were thought to arise from excessive accumulation of Ca^2+^ in the SR, leading to store overload induced Ca^2+^ release. Since evidence supports effects of lysosomal Ca^2+^ release to increase SR Ca^2+^ load, excessive stimulation of this pathway may provoke arrhythmias. In mouse ventricular myocytes addition of 200 nM isoprenaline (40–100 times greater than concentrations used in the experiments above concerning NAADP effects) initially caused an increase in the amplitude of each Ca^2+^ transient, but further exposure led to the appearance of multiple Ca^2+^ transients in response to a single stimulus, indicating arrhythmogenic effects [159]. In subsequent experiments using mag-fura-2 to measure Ca^2+^ levels in the SR, 200 nM isoprenaline caused an initial enhancement in the amplitude of Ca^2+^ depletions in response to electrical stimulation, followed by a progressive development of multiple Ca^2+^ depletions with each stimulus, again indicating an arrhythmogenic effect [160].

Possible arrhythmogenic effects of high concentrations of isoprenaline were investigated in whole hearts from mice lacking TPC2 channels in comparison with WT. Hearts from mice lacking TPC2 channels were substantially less prone to the acute arrhythmogenic effects of 50 nM isoprenaline than WT [79]. Mice were also exposed to prolonged application of isoprenaline by a mini-pump over two weeks. The hearts showed hypertrophy which was less in hearts from mice lacking TPC2 channels. When the hearts were challenged with a burst pacing stimulation protocol, the hearts lacking TPC2 channels were again substantially less prone to arrhythmias [79]. It thus appears that arrhythmias associated with high levels of beta-adrenoceptor stimulation, whether acute or chronic, show a component that is dependent on lysosomal Ca^2+^ release via TPC2 channels leading to excessive accumulation of Ca^2+^ in the SR.

There is some controversy concerning the enzyme responsible for NAADP synthesis in the heart. NAADP has been proposed to act in the heart by a mechanism which complements actions of a second Ca^2+^ mobilizing agent, cADP-ribose [27,30], discussed in more detail below. cADP-ribose is synthesized by an ADP-ribosyl cyclase [16,150], and cardiac actions of this enzyme were enhanced following beta-adrenoceptor stimulation [161]. CD38 is a lymphocyte antigen, and early experiments showed that ADP-ribosyl cyclase and CD38 could catalyse the formation of both Ca^2+^ mobilizing agents, cADP-ribose and NAADP [16,150,162]. Formation of NAADP is thought to occur by a base exchange reaction involving NADP and nicotinic acid [21,151,162,163]. cADP-ribose is synthesized from the substrate NAD [16]. CD38 might be thought of as a subtype of ADP-ribosyl cyclase. Some have argued for a synthesis of NAADP within the lysosome involving a separate enzyme [164]; see discussion in [30].

Cardiac synthesis of NAADP and its role in arrhythmogenic mechanisms was investigated by comparing hearts from mice lacking CD38 with WT [165]. In both cardiac myocyte membrane preparations and intact myocytes synthesis of cADP-ribose and NAADP occurred in WT but was absent in preparations from mice lacking CD38. Fluorescence light microscopy using a specific antibody showed that CD38 was located close to SR in mouse ventricular myocytes and also in rabbit ventricular and atrial myocytes. It was not possible to establish the precise location of CD38 with light microscopy, and it remains possible that CD38 was positioned in the SR membrane. In this context, a preparation routinely used as a source of SR membranes showed ability to synthesize NAADP [165]. This issue and the possible membrane orientation of CD38 are discussed in more detail in [27].

The drug SAN4825 was developed as a potential antiarrhythmic agent specifically targeting synthesis of cADP-ribose [166]. In mouse heart membrane preparations SAN4825 inhibited the synthesis of NAADP as well as cADP-ribose [165]. This occurred at both pH 7.2 (the cytosolic pH) and pH 4.5 (to take into account acidic conditions within the lysosome). Both pH levels were investigated since the precise location and membrane orientation of CD38 in the heart are yet to be established.

Arrhythmogenic effects of high concentrations of isoprenaline were studied both using SAN4825 and mouse hearts lacking CD38 [165]. Pro-arrhythmic effects of 300 nM isoprenaline were greatly reduced in hearts from mice lacking CD38 and by SAN4825.

Synthesis of cADP-ribose by CD38 is also expected to be suppressed in hearts lacking CD38 (and inhibited by SAN4825 in WT hearts) and it seems that this complementary Ca^2+^ mobilizing agent might also be involved in arrhythmias (see below and figure 5).

### (e) Possible role of lysosomal Ca^2+^ release in pacemaker function

Recent observations show that lysosomal function can influence pacemaker function, and following on from the observations in ventricular and atrial muscle, the effects are particularly important during beta-adrenoceptor stimulation. The effects of isoprenaline on spontaneous rate of beating in mouse and guinea pig preparations were reduced both by bafilomycin to block lysosomal function and Ned-19 to antagonize NAADP actions. These effects of isoprenaline were also reduced in preparations lacking TPC2 channels compared to WT. In addition isoprenaline effects on spontaneous rate of beating were less in preparations from mice lacking the NAADP-synthesizing enzyme CD38 than in WT. The observations have been published in abstract form [167] and a full paper has been submitted.

### (f) Lysosomal Ca^2+^ release via two-pore domain channel 1 and ischaemia-reperfusion arrhythmias

Arrhythmias can be initiated during reperfusion following a period of ischaemia. A rapid rise in ROS occurs in the first few minutes of reperfusion. The main source of ROS seems to be mitochondria. MitoQ, an ROS scavenger targeted to mitochondria, is cardioprotective. The relative timing of these processes was investigated in Langendorff hearts using a Ca^2+^-sensitive reporter to measure cytosolic Ca^2+^ [168]. On reperfusion Ca^2+^ waves were observed to propagate across multiple myocytes. A rise in cytosolic Ca^2+^ always occurred before a loss of mitochondrial membrane potential resulting from opening of the mPTP. The coordinated rapid redox changes resulting from reperfusion were observed to occur approximately 2 min before mPTP opening [168].

Ischaemia-reperfusion can be simulated in isolated ventricular myocytes by exposure to a deoxygenated solution with a slightly modified ionic composition containing lactate followed by reoxygenation [169]. This procedure provoked Ca^2+^ waves which were suppressed in the presence of an NAADP antagonist. A substantial increase in cell death was also provoked by simulated ischaemia-reperfusion, and cell death was also suppressed by the NAADP antagonist. In a whole hearts, ischaemia was brought about by occlusion of the left anterior descending cardiac artery for 30 min, followed by reperfusion for 2 h. The size of the resulting cardiac infarct was reduced in animals injected with NAADP antagonist. The involvement of lysosomal TPCs was investigated. Occlusion of the left anterior descending cardiac artery followed by reperfusion caused infarcts that were smaller in TPC1 KO hearts than in WT [169]. In a laser based assay mitochondrial mPTP opening was delayed by exposure to NAADP antagonist. A similar delay in mPTP opening resulted from addition of bafilomycin to suppress lysosomal effects, providing further support for the action of lysosomal Ca^2+^ release on mitochondrial function [169]. The overall conclusion was that NAADP plays a major role in the arrhythmia provoked by reperfusion following ischaemia, and that lysosomal Ca^2+^ release via TPC1 channels close to neighbouring mitochondria is a crucial component in the signalling mechanism. It should also be noted that the EM evidence mentioned above supports a nanodomain between lysosomes and mitochondria with a median separation of 17 nM [13].

Further work confirms the cellular position of lysosomes close to mitochondria, and provides observations consistent with the role of NAADP-mediated lysosomal Ca^2+^ release in arrhythmias associated with ischaemia followed by reperfusion [149]. A novel finding concerns a cardioprotective effect at TPC1 channels that may be clinically important. Both human and mouse tissue showed similar changes under conditions related to ischaemia-reperfusion. In the case of human tissue, atrial biopsies after cardioplegia followed by reperfusion showed an increase in PKARIalpha disulfide state in comparison with minimal PKARIalpha disulfide formation in tissue samples from the same patients before cardiopulmonary bypass. Similarly, left ventricular tissue from mice undergoing transient ligation of the coronary artery showed enhanced PKARIalpha disulfide formation compared with tissue from sham operated hearts. In mouse experiments, disulfide formation did not affect the catalytic activity of PKAIalpha, but did increase binding to A-kinase anchoring protein (AKAP). Binding of PKAIalpha to AKAP caused preferential localization of the holoenzyme to lysosomes. Further experiments were carried out on a strain of mouse in which PKARIalpha was modified to a Cys17Ser configuration which does not support PKARIalpha disulfide formation, and the cells were said to be ‘redox-dead’. For isolated myocytes under tissue culture conditions there is good access of oxygenated solution and the cells are in a highly oxidized state so even under resting conditions WT myocytes showed extensive formation of PKARIalpha disulfide. Myocytes were superfused with a solution lacking both Na^+^ and Ca^2+^, supplemented with the extracellular Ca^2+^ chelator EGTA and tetracaine. On washout of tetracaine spontaneous Ca^2+^ release events occurred in both WT and redox-dead cells, but the fraction of cells showing Ca^2+^ oscillations was much greater in redox-dead cells. The NAADP antagonist, Ned-19, reduced spontaneous Ca^2+^ releases, while bafilomycin to target lysosomes completely abolished this activity. In non-oscillating cells, the SR Ca^2+^ content was normal. It was concluded that Ca^2+^ oscillations on removal of tetracaine were dependent on Ca^2+^ release from lysosomes via TPCs (presumably TPC1 on the basis of the work discussed above), which occurred when PKARIalpha was not localized to the lysosome, as was the case in the redox-dead cells with the Cys17Ser mutation [149]. In isolated hearts with the Cys17Ser mutation and reperfused after global ischaemia, left ventricular pressure was lower than WT. When ventricular tissue was examined the area of damaged tissue in infarcts was greater in Cys17Ser than WT. The harmful effects of ischaemia-reperfusion in Cys17Ser hearts were reduced by Ned-19, so that the left ventricular pressures and infarct sizes were close to WT. Surprisingly, under these conditions the NAADP antagonist seemed to be without a protective effect on WT hearts. Overall the effects of the NAADP antagonist and bafilomycin in these experiments support a role for NAADP-mediated lysosomal Ca^2+^ in harmful effects both in single cells and whole hearts. It was also concluded that disulfide modified PKARIalpha limits harmful effects of reperfusion following ischaemia since it was present in WT cells but was absent after the Cys17Ser modification. It was proposed that PKARIalpha disulfide localized to the lysosome blocks Ca^2+^ release via TPCs, therefore, acting as a ‘gatekeeper’ to protect the heart from the harmful effects of ischaemia [149].

This work highlights an important novel target for new cardioprotective drugs to treat clinical problems associated with the postischaemic heart. The two studies above support the conclusion that the damaging effects of reperfusion following ischaemia depend on Ca^2+^ release from lysosomes via TPC1 channels acting on nearby mitochondria.

## 8. Mitochondria

From the previous section it is clear that mitochondria play a crucial role in the initiation of the particular arrhythmia associated with reperfusion following ischaemia. It seems that any condition that causes a large increase in ROS or disruption of mitochondrial function following opening of mPTP is likely to have dramatic effects on Ca^2+^ cycling and electrical activity of cardiac myocytes [28,170,171].

Mitochondria are a major source of ATP which is essential for cell functions, and mitochondria are abundant in the heart [28,172]. It has been clear for many years that the consumption of ATP by cardiac muscle is higher than any other tissue, and if ATP production is stopped instantaneously the reserves of ATP last less than 1 min [173,174]. As illustrated in figure 2, mitochondria are separated from SR by a nanodomian in which the Ca^2+^ concentration rises and fall with each heartbeat. Interestingly there are important differences between mitochondrial control mechanisms in the heart and in other tissues [29,175]. In the context of the present review, one question concerns the extent to which the extensive network of mitochondria might ‘buffer’ changes in Ca^2+^ concentration that occur in the neighbouring cytoplasm. Experimental evidence shows that changes in mitochondrial Ca^2+^ concentrations are much slower than those in the cytoplasm, so that the rapid and extensive changes in cytosolic Ca^2+^ that occur during Ca^2+^ transients accompanying APs are not closely followed by mitochondrial Ca^2+^ [28]. Indeed changes on the time scale of Ca^2+^ transients are negligible, although slower changes occur, for example when there is a change in the stimulation rate for Ca^2+^ transients [28,176,177]. Additional recent experiments support this view following a detailed investigation of the relationship between the concentration of Ca^2+^ in the mitochondrial matrix ([Ca^2+^]_m_) and cytosolic [Ca^2+^] in the nanodomain between SR and mitochondria [29,175]. Ca^2+^ enters the mitochondrion via the mitochondrial uniporter complex (MCU_cx_), as shown schematically in figure 2. There is a low and constant number of MCU_cx_ in the heart (only about 5–15 in each mitochondrion) and these serve as ‘gatekeepers’ to prevent excessive Ca^2+^ influx and ‘overload’ of [Ca^2+^]_m_ during high Ca^2+^ transients [29,175]. ATP production is controlled by [Ca^2+^]_m_ and the voltage across the inner mitochondrial membrane Δ*Ψ*_m_. During a period of prolonged quiescence when cytosolic Ca^2+^ concentration is stable at around 100 nM, the [Ca^2+^]_m_ is slightly higher. The local cytosolic Ca^2+^ concentration can reach as high as 10 µM when the cell is stimulated to fire Ca^2+^ transients accompanying APs. However, initiation of APs after a period of quiescence causes only a slow steady rise in [Ca^2+^]_m_ to reach a new stable level over a period of many minutes [29,175]. Beta-adrenoceptor stimulation causes an increase in the amplitude of cytosolic Ca^2+^ transients and leads to a slow further rise in [Ca^2+^]_m_. It, therefore, appears that the frequency and amplitudes of Ca^2+^ transients determine [Ca^2+^]_m_ and consequently ATP production.

Although under ‘normal’ conditions [Ca^2+^]_m_ is stable, and the behaviour of mitochondria is matched to function this is clearly not the case (as discussed above) under conditions in which there is a substantial increase in ROS and opening of mPTP. In addition there are other conditions in which mitochondrial mechanisms appear to contribute to disturbances of electrical activity including generation of DADs [178,179], and cardiac alternans [180]. Further discussion of the importance of mitochondrial–SR junctions for arrhythmias and other cardiac pathophysiology can be found in [181].

In the context of the major themes of this review it may be questioned how NAADP can exert effects on SR that are beneficial (at least until stimulation becomes excessive) while effects on mitochondria are harmful. The physiological conditions for the two effects are dramatically different but this may not be the only factor underlying the difference in effects. This topic has been discussed extensively elsewhere [27]. It remains possible that even under physiological conditions there might be simultaneous effects of NAADP on mitochondria and SR when NAADP mediates lysosomal Ca^2+^ release. It has been argued that it is hard to see how evolutionary pressures would result in lysosomal Ca^2+^ release via TPC1 channels only having harmful effects on mitochondria under conditions of ischaemia-reperfusion [27]. It is conceivable that there might be an additional beneficial effect of lysosomal Ca^2+^ release via TPC1 channels on mitochondrial function which is yet to be explored experimentally. Another issue concerns whether the distribution of TPC1 and TPC2 channels on the lysosomal membrane is uniform or whether TPC1 and TPC2 are arranged to face their targets. Evidence also supports the proposal that the relative expression of TPC1 and TPC2 channels varies with the developmental stage in the endolysosomal system [33]. On the basis of this scheme it seems possible that the organelles expressing TPC1 channels which are responsible for ischaemia-reperfusion arrhythmias might be at an earlier stage of development compared with organelles expressing TPC2 channels involved in regulation of SR Ca^2+^ content. This would allow separate activation of different sets of related organelles carrying either TPC1 or TPC2. These questions remain for future study.

## 9. Calcium mobilizing agents

### (a) Nicotinic acid adenine dinucleotide phosphate

NAADP is widely recognized as a Ca^2+^ mobilizing agent with diverse actions throughout the body [16,21,22,151]. Evidence that NAADP also acts in the heart as an important Ca^2+^ mobilizing agent was presented above in the context of activation of lysosomal Ca^2+^ release to result in modulation of SR and mitochondrial function. Further discussion of these cardiac actions can be found in [12,30] and [27]. A recent review also considers possible intermediary binding proteins [33].

### (b) cADP-ribose

cADP-ribose is a Ca^2+^-mobilizing agent which promotes Ca^2+^ release from endoplasmic reticulum via an action at RyR2s in many cell types [16,151]. In the heart, photoreleased cADP-ribose increased the amplitude of Ca^2+^ transients accompanying APs, without an effect on triggering L-type Ca^2+^ currents and without an effect on the amount of Ca^2+^ loaded into the SR as assessed from the Ca^2+^ release in response to a high concentration of caffeine [182]. It was concluded that a least for the first few minutes of exposure the primary effect of cADP-ribose was to act at the RyR2 to increase the likelihood of Ca^2+^ release from the SR. This interpretation was supported by Prakash *et al.* [183], and earlier experiments using 8-amino-cADP-ribose, an antagonist of cADP-ribose [81,184] and [80].

The above conclusion remains controversial. On the basis of observations with caffeine, and related theoretical arguments, it has been asserted that all agents acting at RyR2s to increase Ca^2+^ release from the SR cannot cause a maintained effect on the amplitude of Ca^2+^ transients because of a compensatory mechanism in which the amount of Ca^2+^ in the SR declines to exactly match the effect of enhanced release [185]. The experimental observations with cADP-ribose and other work discussed below contradict these theoretical arguments. Actions of cADP-ribose differ substantially from caffeine, which is membrane permeant and crosses not only the surface membrane but also the SR. By contrast, cADP-ribose is a polar molecule showing low ability to cross cell membranes. The mechanism of action of caffeine is thought to involve a reduction of the threshold for activation by luminal Ca^2+^ with little effect on the threshold for cytosolic Ca^2+^ [186], but see [187]. cADP-ribose is thought to promote Ca^2+^ release from the SR via RyR2s by a cytosolic action which may be related to FKBP12.6, a protein associated with RyR2 [188]. There may be an intermediary binding protein [27]. Another difference is that while both agents increase Ca^2+^ spark frequency, SR Ca^2+^ content was reduced by caffeine since spark amplitude declined, but spark amplitude remained unchanged with cADP-ribose [182]. Caffeine causes Ca^2+^ release from the SR at a range of concentrations [189], and high concentrations cause complete emptying of SR Ca^2+^. Caffeine may, therefore, have a diastolic effect to promote SR Ca^2+^ loss which is not shared by cADP-ribose. 8-amino-cADP-ribose blocked the effect of photoreleased cADP-ribose on Ca^2+^ transients accompanying APs, but did not block the response to caffeine [81]. The effect of 8-amino-cADP ribose to reduce the amplitude of Ca^2+^ transients accompanying APs was maintained and did not show compensatory changes with time [81]. Both cADP-ribose and NAADP increased the amplitude of Ca^2+^ transients accompanying APs, but for observations over several minutes cADP-ribose was without effect on SR Ca^2+^ content while NAADP caused a substantial increase. The above evidence supports substantial differences between the actions of cADP-ribose and caffeine.

An important aspect of the actions of cADP-ribose was that the effects were markedly temperature dependent so that actions at room temperature were negligible while substantial effects occurred close to body temperature [190].

Questions remain about the exact mechanism of action of cADP-ribose at RyR2s. Early observations show that cADP-ribose did not increase Ca^2+^ release via RyR2s in experiments in planar lipid bilayers [191–193] and SR microsomes [193]. However, later experiments showed that cADP-ribose increased Ca^2+^ spark frequency in permeabilized ventricular myocytes from WT mice but not in FKBP12.6 knockout mice. cADP-ribose displaced FKBP12.6 from mouse cardiac SR vesicles. Evidence in rat pancreatic islets [194], arterial and tracheal smooth muscle [195,196] and adrenal chromaffin cells [197] also supports cADP-ribose action at FKBP12.6. Nevertheless, this mechanism remains controversial and a well-balanced discussion of the issue can be found in [198].

FK506 also increased the amplitude of Ca^2+^ transients accompanying APs under conditions in which the SR Ca^2+^ load and Ca^2+^ currents were unchanged [199]. These observations provide further experimental evidence against the theoretical argument mentioned above that agents with their principal effect on RyR2s cannot cause a maintained increase on the amplitude of Ca^2+^ transients.

Even if effects of cADP-ribose over the first few minutes were simply on RyR2s to promote Ca^2+^ release from the SR, there might be additional indirect effects resulting from the consequent rise in cytosolic Ca^2+^, perhaps in localized regions. In cardiac homogenates and permeabilized cells the major effect of cADP-ribose was to increase Ca^2+^ uptake into the SR by SERCA [200]. Following these observations, mechanisms of action of cADP-ribose were reinvestigated [201]. Again the amplitudes of contractions and Ca^2+^ transients accompanying APs were increased when cADP-ribose was applied either by patch pipette or photorelease in guinea pig ventricular myocytes. In each case there was no increase in SR Ca^2+^ assessed from the response to a high concentration of caffeine (measured either as the integral of NCX current or fluo-4 fluorescence). In rat ventricular myocytes permeabilized with saponin, application of cADP-ribose for 30 s and 3 min increased Ca^2+^ spark frequency without a change in SR Ca^2+^ content (assessed from the response to caffeine). There was also no change in spark amplitude or decay time providing further independent evidence that SR Ca^2+^ content was not increased [201]. However, at 10 min there was an enhanced response to caffeine and increased spark amplitude indicating an increased SR Ca^2+^. There was also a quickening of spark decay time which was taken to result from increased activity of SERCA. This may have arisen as a secondary consequence of a prolonged increase in spark frequency with possible effects on PLB/SERCA [201].

In summary, effects of cADP-ribose over at least the first several minutes lead to an increase in the amplitude of Ca^2+^ transients associated with APs by a cytosolic action at RyR2s without an increase in SR Ca^2+^ content [201]. Prolonged exposure to cADP-ribose has additional actions involving SR Ca^2+^ uptake. Under physiological conditions, cADP-ribose probably acts in concert with NAADP, when the enhanced uptake of Ca^2+^ into the SR resulting from NAADP-mediated effects will complement the actions of cADP-ribose on Ca^2+^ release via RyR2s (see [30] and [27] for more detailed discussion of these complementary actions).

### (c) cADP-ribose and arrhythmias resulting from excessive stimulation of beta-adrenoceptors

In the section above on DADs, it was mentioned that 8-amino-cADP-ribose, an antagonist of cADP-ribose, suppressed arrhythmias brought about by high levels of beta-adrenoceptor stimulation (figure 5; [81]). It was also noted that beta-adrenoceptor stimulation caused an increase in the synthesis of both cADP-ribose and NAADP [157]. In addition, evidence presented above supports CD38 as the primary enzyme in the heart catalysing synthesis of both cADP-ribose (with NAD as substrate) and NAADP (requiring a base change mechanism involving NADP and nicotinic acid). The reduction of isoprenaline-induced arrhythmia in mouse hearts lacking CD38 compared to WT [165] may reflect a reduction in synthesis of both cADP-ribose and NAADP.

### (d) I nositol trisphosphate

IP_3_ is a Ca^2+^ mobilizing agent of major importance in the heart. Evidence concerning its actions is very well described elsewhere [202] and only a brief summary will be presented here. Early observations showed that IP_3_ can provoke Ca^2+^ release from the SR [70]. IP_3_ exerts particularly important effects in atrial myocytes where expression of type II IP_3_ receptors is much higher than in ventricular myocytes. IP_3_ acts on junctional SR beneath the plasmalemma to increase the amplitude of Ca^2+^ transients accompanying APs [203]. Effects of IP_3_ on short term function of ventricular myocytes were minor in comparison to those in atrial cells, but long-term effects occur in both cell types as a result of changes in protein synthesis following nuclear actions of IP_3_ [204].

Further experiments showed functional effects of IP_3_ in atrial myocytes [205,206]. Endothelin activated the IP_3_ pathway, and could cause arrhythmias [206]. Endothelin effects did not occur in atrial myocytes from mice lacking Type II IP_3_R [207].

Alpha-1 adrenoceptors also activate the IP_3_ pathway to increase the amplitude of Ca^2+^ transients accompanying APs, and in experiments on cat atrial myocytes the actions involved endothelial nitric oxide synthase (eNOS) and production of nitric oxide (NO) [208]. In guinea pig atrial myocytes alpha-receptor-mediated effects to increase Ca^2+^ transients seemed to depend on a novel signalling pathway involving IP_3_ and Ca^2+^-stimulated adenylyl cyclases, AC1 and AC8, which were first described in heart by Mattick *et al.* [142]. Photoreleased IP_3_ increased the amplitude of Ca^2+^ transients accompanying APs, and the effects were suppressed by the AC inhibitor MDL12,330A and by the PKA inhibitor H89. It was suggested that IP_3_ caused Ca^2+^ release from the SR which acted on neighbouring Ca^2+^-stimulated AC1 and AC8 to provoke actions via the cAMP/PKA pathway. Under the conditions of the experiments the NO signalling pathway seemed not to be a major contributor since effects of photoreleased IP_3_ on Ca^2+^ transient amplitude were little if at all affected in the presence of L-NAME to inhibit eNOS or ODQ to inhibit guanylyl cyclase [143].

There also appears to be a role for IP_3_ signalling in pacemaker function by actions that involve Ca^2+^ handling by the SR [146,209,210].

Reviews of IP_3_ actions in the heart can be found in [211–213] and [202].

## 10. Revisiting the importance of Ca^2+^/calmodulin-dependent kinase II

Previously well-known actions of CaMKII were summarized and discussed in an early section of this review. In subsequent sections evidence has been presented for the involvement of CaMKII in the signalling pathway linking lysosomal Ca^2+^ release to enhanced SR Ca^2+^ uptake, and in effects mediated by the calcium mobilizing agents cADP-ribose and NAADP. cADP-ribose is thought to act at RyR2s, though this remains controversial. Since CaMKII can phosphorylate RyR2s such phosphorylation might perhaps be a requirement for cADP-ribose action, but additional work is needed to test this proposal. CaMKII was thought to be necessary for cADP-ribose actions in pancreatic islets [214]. The observations concerning a requirement for CaMKII for the cardiac actions of NAADP seem more secure since this was observed in ventricular and atrial myocytes, and both autocamtide-2-related inhibitor peptide and KN-93 have been shown to suppress NAADP actions (while the inactive KN-92 was shown to be without effect) [79]. In addition, an amplification mechanism involving CaMKII phosphorylation of PLB/SERCA accounts for the observed substantial increase in SR Ca^2+^ content following NAADP-induced Ca^2+^ release from lysosomes (which occupy a much smaller fraction of the cell than SR) [79,147,148].

As outlined above, the arrhythmogenic effects of high levels of beta-adrenoceptor stimulation include actions of NAADP and cADP-ribose following an increase in their synthesis by CD38, and CaMKII is expected to be involved in the effects of both these calcium mobilizing agents during arrhythmias resulting from excessive beta-adrenoceptor stimulation. This evidence must be taken into account to provide a full explanation of the contribution of CaMKII to arrhythmogenic mechanisms. It is also possible that these more recently discovered mechanisms might contribute to the linkage of PKA and CaMKII effects discussed in [215].

## 11. Summary

The evidence presented here provides a compelling case for the influence of intracellular organelles on electrical activity recorded across the surface membrane. The operation of NCX plays a central role in linking events within the cell to changes in surface membrane electrical activity. Most frequently NCX causes depolarizations resulting from increases in subsarcolemmal Ca^2+^ concentrations with subsequent Ca^2+^ extrusion, although there may be additional roles for ion channels activated or inhibited by cytosolic Ca^2+^.

The SR exerts a dominant influence in initiating arrhythmogenic events under pathological conditions in all regions of the heart. Ca^2+^ released from the SR can contribute to EADs, and is crucially important for the generation of DADs and alternans. Under ‘normal’ physiological conditions, the SR plays a role in the timing of the natural pacemakers in the SA and AV nodes.

Lysosomes can also have important effects on cardiac function. The Ca^2+^ mobilizing agent, NAADP is synthesized by CD38 and acts on lysosomes to release Ca^2+^ via TPC2 channels into a nanodomain between the lysosome and SR membranes. Evidence supports the hypothesis that Ca^2+^ released into this nanodomain activates CaMKII leading to phosphorylation of PLB and enhancement of Ca^2+^ uptake into the SR by SERCA. This results in an increase in the amplitude of Ca^2+^ transients accompanying APs. In the heart the synthetic enzyme, CD38, is located at or close to the SR. Synthesis of NAADP and therefore its effects are enhanced following beta-adrenoceptor stimulation. Excessive stimulation of this pathway, whether acute or chronic, is arrhythmogenic. A separate pathway involving lysosomes is a dominant influence in arrhythmias caused by reperfusion after a period of ischaemia. In this case NAADP causes Ca^2+^ release from lysosomes via TPC1 channels into a nanodomain between lysosomes and mitochondria, and the arrhythmia results from an interaction between the three intracellular organelles, lysosomes, mitochondria and SR. A protective effect of PKAIalpha has been identified which is thought to result from inhibition of Ca^2+^ release via TPC1 channels in the lysosomal membrane. Mitochondria are also involved in other arrhythmogenic events.

In addition to NAADP, two other Ca^2+^ mobilizing agents, cADP-ribose and IP_3_ can contribute to the generation of arrhythmias under appropriate conditions. cADP-ribose is also synthesized by CD38, with production enhanced following stimulation of beta-adrenoceptors. Excessive stimulation again causes arrhythmias which can be suppressed by an antagonist of cADP-ribose. cADP-ribose is thought to act on RyR2s via FKBP12.6, but this remains controversial.

CaMKII plays a pivotal role in coordinating the functions of different intracellular organelles and their interactions with the surface membrane.

The evidence presented here strongly supports the proposal that intracellular organelles play diverse roles in controlling the timing mechanisms which determine heart rhythms and initiate cardiac arrhythmias.

## Data Availability

This article has no additional data.
